# Editorial: Nutrition, inflammation and oxidative stress in obstetrics and gynecology

**DOI:** 10.3389/fnut.2025.1749449

**Published:** 2025-12-04

**Authors:** Małgorzata Mizgier, Dorota Formanowicz, Justyna Opydo-Szymaczek, Maria Luisa Mizgier

**Affiliations:** 1 Department of Sports Dietetics, Chair of Dietetics, Faculty of Health Sciences, Poznan University of Physical Education, Poznan, Poland; 2 Chair and Department of Medical Chemistry and Laboratory Medicine, Poznan University of Medical Sciences, Poznan, Poland; 3 Chair and Department of Pediatric Dentistry, Poznan University of Medical Sciences, Poznan, Poland; 4 Faculty of Dentistry, Universidad de Los Andes, Santiago, Chile; 5 Center for Biomedical Research and Innovation (CIIB), Universidad de Los Andes, Santiago, Chile

**Keywords:** nutrition, oxidative stress, inflammation, women's health, obstetrics and gynecology, reproductive disorders, metabolic dysregulation, lifestyle interventions

## 1 Introduction

Oxidative stress (OS) and chronic low-grade inflammation are pivotal in the pathophysiology of numerous reproductive and metabolic disorders from infertility, pregnancy complications to menopausal transitions. Nutrition, lifestyle, and environmental exposures strongly influences these processes, shaping redox homeostasis, immune responses, and vascular function. Dietary antioxidants, pro-inflammatory dietary patterns, and metabolic dysregulation form a complex network linking oxidative imbalance to hormonal and vascular dysfunction.

The aim of this Research Topic was to elucidate how nutritional factors modulate inflammation and oxidative stress across obstetric and gynecologic conditions, combining mechanistic, observational, and interventional perspectives. These multidimensional relationships, 19 peer-reviewed papers were included, encompassing observational studies, reviews, cross-sectional studies and mechanistic analyses.

## 2 Overview of the contributions

### 2.1 Oxidative stress, inflammation, and pregnancy outcomes

Several studies addressed metabolic and micronutrient imbalances as modulators of pregnancy outcomes.

Mo et al. showed that higher maternal serum ferritin concentrations in early and late gestation were independently associated to hypertensive disorders of pregnancy in a longitudinal cohort, consistent with ferritin's dual role as an iron-storage and acute-phase inflammatory protein. Although direct measurements of oxidative stress and inflammation were not performed, ferritin, as an acute-phase reactant, may indirectly reflect oxidative and inflammatory processes relevant to adverse pregnancy outcomes.

Li, Su et al. demonstrate that an elevated non-HDL to HDL cholesterol ratio (NHHR) in early to mid-pregnancy predicts an increased risk of gestational diabetes mellitus (GDM), supporting its potential use as an early metabolic predictor.

While Zheng et al. and Li, Zhu et al. reviewed the contributions of vitamin D deficiency and nutrient metabolic reprogramming to early-onset pre-eclampsia. Summarizing evidence for its anti-inflammatory and antioxidant roles in placental function and how alterations in carbohydrate, lipid, amino acid and glycan metabolism relate to oxidative stress, trophoblast autophagy, inflammation and vascular dysfunction.

Together, these studies underscore that metabolic, lipid, and micronutrient disturbances serve as both markers and mediators of pregnancy-related oxidative stress and inflammation, highlighting their potential relevance for early risk assessment and the development of targeted nutritional strategies.

### 2.2 Endometriosis and estrogen-dependent gynecological disorders: redox-immunometabolic pathways

Endometriosis, a chronic inflammatory disorder with systemic alterations, was discussed across several articles.

Chen et al. associated higher serum uric acid levels with increased risk endometriosis using NHANES data from 1999 to 2006. This finding showed that women in the highest quartile had an approximately 2.3-fold higher risk of endometriosis compared to those in the lowest quartile.

Whereas, Yu et al. found a protective effect of higher composite dietary antioxidant index (CDAI) values, identifying a protective dose–response relationship in a US adult female population.

Zhou et al. reviewed anti-inflammatory and antioxidant dietary interventions, highlighting the benefits of plant-based, polyphenol- and omega-3-rich diets.

Conversely, Zhang, Li, Zhang et al. identified elevated vitamin B6 and calcium levels as potential risk factors and suggesting a role for micronutrient imbalances in oxidative and hormonal dysregulation, and Li, Xie et al. using Mendelian randomization, found no significant genetic causal between diet-derived circulating antioxidants and endometriosis risk.

Fu et al. linked high sugar-sweetened, energy, and alcoholic drinks to the occurrence of endometrial polyps. Their findings indicated that frequent consumption of such beverages may contribute to oxidative and inflammatory changes in the endometrium, supporting the role of dietary factors in the pathogenesis of estrogen-dependent gynecological disorders.

These findings collectively underscore the multifaceted role of antioxidant status, micronutrient balance, and dietary quality in modulating oxidative and inflammatory pathways. Optimizing nutritional profiles may help mitigate the progression of estrogen-dependent gynecological disorders such as endometriosis.

### 2.3 Polycystic ovary syndrome and metabolic dysregulation

Polycystic ovary syndrome (PCOS), a paradigm of metabolic–reproductive interplay, was discussed by Mizgier et al., who reported that in adolescent girls with PCOS, both hyperandrogenism and low-grade inflammation were inversely related to bone formation markers, suggesting detrimental effects on bone metabolism independent of body weight. The authors highlighted that oxidative stress may impair, whereas antioxidant capacity may support, bone formation in this population.

Ghavami et al. showed that higher dietary insulin index and load correlate with diminished ovarian reserve, underscoring the deleterious impact of hyperinsulinemia on ovarian function. These findings suggest that reducing dietary insulin load may help preserve reproductive health by mitigating metabolic and inflammatory stress.

These insights converge on metabolic optimization through lifestyle and dietary interventions as a strategy to reduce oxidative and inflammation burden, and hormonal imbalances, improving both ovarian and bone health in women with metabolic–reproductive disorders.

### 2.4 Nutritional and hormonal-inflammatory interactions beyond reproduction

Several articles examined how nutritional modulation influences systemic inflammatory and endocrine pathways in non-pregnant women. Zhang, Li, Yang et al. demonstrated an inverse association between serum iron levels and Hashimoto's thyroiditis in US women of reproductive age, suggesting that iron deficiency may drive autoimmune and oxidative stress responses.

Hu et al. showed that low serum 25(OH)D (vitamin D) concentrations increase ovarian cancer risk, but not with other female-specific cancers. The findings highlight the potential antioxidant and immunoregulatory role of vitamin D in ovarian carcinogenesis.

Tain and Hsu reviewed maternal consumption of nutritive and non-nutritive sweeteners and its programming effects on offspring hypertension, highlighting oxidative stress, dysregulated nutrient-sensing pathways, and renin–angiotensin system activation as potential mechanisms of developmental programming.

Wierzejska et al. reported adequate maternal and umbilical cord 25(OH)D concentrations in twin pregnancies, likely due to supplementation, finding strong mother–newborn correlations and low rates of deficiency, while highlighting the impact of environmental and lifestyle factors on vitamin D status.

Mo et al. reported a significant association between the dietary inflammatory index (DII) and type 2 diabetes risk in US women, while Liu et al. confirmed that higher CDAI levels inversely relate to metabolic syndrome prevalence in women. The study also revealed that estrogen levels were negatively correlated with the incidence of metabolic syndrome, highlighting the interplay between oxidative balance, hormonal regulation, and metabolic health.

Together, these studies highlight how nutritional status and dietary composition influence systemic inflammation and endocrine function beyond reproductive health. Micronutrient deficiencies, unbalanced dietary patterns, and excessive intake of additives or sweeteners emerge as important modifiers of oxidative and metabolic homeostasis in women. These findings support the growing recognition that optimizing diet quality may contribute to the prevention of inflammatory and metabolic disorders throughout the female lifespan.

### 2.5 Metabolic–inflammatory links to periodontal health

Oral health, an often-overlooked marker of systemic inflammation, was also explored. In this context, periodontitis—a major inflammatory disease of the oral mucosa, is epidemiologically associated with other chronic inflammation-driven disorders.

Wang et al. associated the triglyceride–glucose index and body roundness index with periodontitis risk, suggesting a metabolic–inflammatory bridge between oral and systemic health.

Although behavioral determinants such as oral hygiene were not assessed, this large-scale cross-sectional analysis provides valuable epidemiological insight and supports the growing evidence linking metabolic dysregulation with inflammatory periodontal disease.

This finding highlights the increasing recognition of oral health as a vital component of systemic metabolic and inflammatory homeostasis.

## 3 Emerging insights and future directions

This Research Topic illustrates the interdisciplinary integration of nutrition, endocrinology, obstetrics, and oral-systemic health. Nutritional interventions can modulate oxidative stress and inflammatory cascades, with implications extending from cellular metabolism to reproductive outcomes and oral health.

Clinically, these findings underscore the potential of diet-based strategies as adjunctive therapies in obstetric and gynecological care. From a translational perspective, identifying redox and inflammatory biomarkers responsive to nutrition may help tailor interventions, inform personalized nutrition strategies in women's health, and also predict clinical outcomes.

Future research should employ longitudinal, omics-based, and interventional designs to clarify causality and evaluate the synergic effects of diet, microbiota, lifestyle, and environmental exposures.

## 4 Concluding remarks

Collectively, the 19 contributions in this Research Topic strengthen the concept that nutrition acts as both mediator and modifiable determinant of oxidative–inflammatory balance across women's life stages integrating nutritional science into obstetric and gynecologic research is essential to advancing precision prevention and care for women worldwide.

